# Supplementary material for: Government health care worker training needs for intestinal schistosomiasis morbidity management
Source: PLoS Negl Trop Dis. 2026 Jun 8;20(6):e0014419. doi: 10.1371/journal.pntd.0014419 (PMC13268167; doi:10.1371/journal.pntd.0014419)
Supplement: S1 Text — (DOCX) [file pntd.0014419.s001.docx]

**S1 Text: An example of a hepatosplenic case management by an expert clinician**

The patient had experienced progressive abdominal distension over a two-year period, which had worsened in the preceding three days, prior to observation. On general examination, findings were documented and monitored over several days to assess the evolving clinical picture. Laboratory results showed neutropenia, with additional tests- liver function (Aspartate Aminotransferase (AST)/ alanine transaminase (ALT) tests), as well as renal function parameters performed to evaluate possible organ involvement. The management plan included emergency blood transfusion, administration of furosemide, tranexamic acid, propranolol, and spironolactone, with insertion of a urethral catheter to monitor urine output and assess renal function. The clinician emphasized the role of a plant-based protein diet to reduce hepatic metabolic load while maintaining adequate nutrition. A plasma expander was used to address hypovolemia. Notably, the patient passed melena stools predominantly at night, a finding discussed in relation to portal hypertension and variceal bleeding risk. The clinician also highlighted that the AST-to-Platelet Ratio Index (APRI) score can be used to assess liver fibrosis severity. Ultimately, a review of the referral system revealed that the patient had been lost to follow-up.
