# Supplementary material for: Government health care worker training needs for intestinal schistosomiasis morbidity management
Source: PLoS Negl Trop Dis. 2026 Jun 8;20(6):e0014419. doi: 10.1371/journal.pntd.0014419 (PMC13268167; doi:10.1371/journal.pntd.0014419)
Supplement: S2 Text — (DOCX) [file pntd.0014419.s002.docx]

**S2 Text: Pre-set facilitator questions used during the workshops to guide discussions on mapping out patient pathways**

1. What are the first steps for patient triage?
2. What would be the provisional diagnoses?
3. How do you make this decision? (name any tests/examinations you would you do?)
4. Who makes this decision? Who works with who?
5. What would be the differential diagnoses?
6. How do you make this decision? (name any tests/examinations you would you do?)
7. Who makes this decision? Who works with who?
8. What are some key issues that persons quality of life/ what are the patients concerns?
9. What can you address with resources available?
10. What can you not address with resources available?
11. Should the patient be referred? If so what would be the reason?
12. What are some solutions if you cannot refer the patient?
13. How do you communicate the findings with the patients?
14. Do you come across any issues here?
15. Would you follow up with the patients?
16. How would you contact them?
17. How long after would you follow them up?
18. Are there any issues with patient follow up?
19. Is there anything you are uncertain of?
