# Supplementary material for: Government health care worker training needs for intestinal schistosomiasis morbidity management
Source: PLoS Negl Trop Dis. 2026 Jun 8;20(6):e0014419. doi: 10.1371/journal.pntd.0014419 (PMC13268167; doi:10.1371/journal.pntd.0014419)
Supplement: Attachment — Submitted filename: Response to reviewers.docx [file pntd.0014419.s011.docx]

Reviewer Comments Point by Point Response

Reviewer #1

Methods

No concerns to raise under methodology
Ethical and regulatory requirements adequately met.

Results

-Does the analysis presented match the analysis plan?
-Are the results clearly and completely presented?
-Are the figures (Tables, Images) of sufficient quality for clarity?

Reviewer response: Yes

Conclusions:

-Are the conclusions supported by the data presented?
-Are the limitations of analysis clearly described?
-Do the authors discuss how these data can be helpful to advance our understanding of the topic under study?
-Is public health relevance addressed?

Reviewer response: Yes

Editorial and Data Presentation Modifications?

Highly relevant topic -manuscript well presented.

Summary and General Comments

This manuscript addresses a highly relevant topic of schistosomiasis morbidity, which remains under-reported. The gap between infection-focused interventions (especially mass treatments) and the management of chronic morbidity within routine health systems persists. Using a qualitative needs assessment across three endemic districts in Uganda, the authors provide important insights into health worker knowledge gaps, fragmented patient pathways, and systemic constraints in morbidity management. The topic is timely and aligns well with the evolving global agenda to move beyond preventive chemotherapy toward integrated morbidity management and health system strengthening.

***Author Response:***

*We sincerely thank Reviewer #1 for their time, thorough evaluation, and valuable feedback on our manuscript. We are encouraged that the reviewer found our objectives clear, our methodology sound, and our conclusions regarding the management of chronic schistosomiasis morbidity, and the existing gaps within primary health care settings well supported by the evidence.*

Reviewer #2

***Author Response:***

*We sincerely thank Reviewer #2 for their assessment of our manuscript and for recognizing its relevance to the management of schistosomiasis morbidity. We have carefully considered the reviewer’s suggestions and revised to improve the clarity of the manuscript. Below, we provide detailed, point-by-point responses to the specific points raised in the review.*

Methods

***Comment 1***

- The manuscript titled "Government health care worker training needs for schistosomiasis morbidity
  management" is a good paper that highlight important thematic area about morbidity management of schistosomiasis infection, looking at training and capacity building needs of the health system to be able provide the needed care for those affected. The manuscript is well written with clearly articulated objectives, and it employed the appropriate study design. However, some sentences are too long, and need broken into 2 sentences while some need rephrasing.

***Author Response:***

*We thank the reviewer for these comments. We have carefully reviewed the entire manuscript and revised it accordingly by breaking up overly long sentences and rephrasing others to improve clarity and readability.*

Results

The analysis presented match the analysis plan and the result were clearly and presented in detailed. The tables presented are okay.

Conclusions

The conclusions were well supported by data presented and the limitation of the analysis were described. In addition, authors explain how their findings can be use in health workers need assessment in other schistosomiasis endemic regions.

Editorial and Data Presentation Modifications?

***Comment 2***

- The manuscript is good and relevant, but the authors need to work on some statements, some are too long or need rephrasing for clarity.

***Author Response:***

*We thank the reviewer for these comments. We have carefully reviewed the entire manuscript and revised it accordingly by breaking up overly long sentences and rephrasing others to improve clarity and readability.*

Summary and General Comments

The manuscript is relevant and useful for ministry of health of schistosomiasis endemic community that wish to conduct need assessment for management of schistosomiasis morbidity at all levels of health care.

Additional Comments

***Comment 1***

- Please add reference to support this statement under the Introduction section ‘Schistosomiasis is a leading cause of disability and likely mortality in sub-Saharan Africa (SSA) (Line 75)’.

***Author Response****:*

*References have been added to support the statement.*

***Comment 2***

- Can the statement in lines 190-193, be rephrased for better clarity. Consider this, Participants were then divided into groups of 5-7 people to map out the patient pathways for an assigned case study. Each group were moderated by the central Ministry of health staff or SchistoTrack team members

***Author Response****:*

*We thank the reviewer for this suggestion. The statement has been rephrased for improved clarity, as recommended.*

***Comment 3***

- The word 'as' is missing in lines 286-287

***Author Response****:*

*We thank the reviewer for pointing this out. The word ‘as’ has been added as suggested.*

***Comment 4***

- Add the word 'management' before the word "and" (line 431).

***Author Response****:*

*We thank the reviewer for this suggestion. The word ‘management’ has been added before the word’ and’, as recommended.*

***Comment 5***

- Can you rephrase or break the sentence for better clarity and flow (Lines 452-455)

***Author Response****:*

*We thank the reviewer for this suggestion. The section has been rephrased to read as follows: ‘Case management and patient pathways rely on passive case detection, which is typically initiated when patients seek care based on various factors. The most appropriate management strategy depends on the healthcare setting, the level of available clinical expertise, and the patient’s social and clinical context.’*

***Comment 6***

- Replace ‘more’ with 'additional' (Line 456)

***Author Response****:*

*We thank the reviewer for this suggestion. The word ‘more’ has replaced with ‘additional’, as recommended.*

***Comment 7***

- Replace ‘for’ with 'on' (Line 456)

***Author Response****:*

*We thank the reviewer for this suggestion. The word ‘for’ has replaced with ‘on’, as recommended.*

***Comment 8***

- Kindly rephrase this statement as 'Our study further corroborates these findings as many patients fail to reach referral centre due to high cost of transportation’ (Lines 457-458)

***Author Response****:*

*We thank the reviewer for this suggestion. The statement has been rephrased for improved clarity, as recommended.*

***Comment 9***

- Please rephrase (lines 469-470) for clarity and better flow.

***Author Response****:*

*We thank the reviewer for this suggestion. The lines have been rephrased to read as follows: ‘There is a need for country-specific guidelines that clearly outline referral pathways and their integration with other health service platforms’.*

***Comment 10***

- Please break the sentence to enhance clarity and better flow (Lines 478-481)

***Author Response****:*

*We thank the reviewer for this suggestion. The lines have been rephrased to read as follows: ‘They also highlighted coordination with better-equipped stakeholders, including private clinics, as a practical solution for managing intestinal schistosomiasis-related morbidity. Such collaboration could help build capacity in public facilities that currently lack adequately trained personnel and essential tools’.*

Reviewer #3

***Author Response***

*We sincerely thank Reviewer #3 for their assessment of our manuscript and for recognizing its relevance to the management of intestinal schistosomiasis morbidity in Uganda and in other endemic settings. We have carefully considered the reviewer’s suggestions and revised our manuscript.*

*Below, we provide detailed, point-by-point responses to the specific points raised in the review.*

Methods

***Comment 1***

- Are the objectives of the study clearly articulated with a clear testable hypothesis stated? The objectives are stated in different paragraphs of the manuscript: in the introduction and in the study design and participant recruitment. The objectives should be stated in the introduction paragraph and rephrased (What is general objective? and the specific objectives?). A clear testable hypothesis is not required since it is a qualitative study.

***Author Response:***

*We thank the reviewer for this helpful suggestion. We have now revised the Introduction section to clearly state both the general and specific objectives in a single paragraph. The general objective is explicitly distinguished from the specific objectives, which have been rephrased for clarity.*

Is the study design appropriate to address the stated objectives? YES
Is the population clearly described and appropriate for the hypothesis being tested? There is no need of hypothesis to be tested. Study participants are clearly described

Is the sample size sufficient to ensure adequate power to address the hypothesis being tested? There is no hypothesis to be tested. To get information from a qualitative study the sample size is sufficient. However as stated in the limitations, the workshops did not include all types of health workers in the study area and the generability of the findings is limited.

Were correct statistical analysis used to support conclusions? Statistical analyses are not required since it is a qualitative study.

Are there concerns about ethical or regulatory requirements being met? The study obtained ethics approval and there is no concern identified.

***Comment 2***

- Row # 203-205: "After the patient pathway...inadequacy to their colleagues" should be removed and used in the discussion paragraph.

***Author Response****: Thank you for the suggestion. We have deleted the phrase “inadequacy to their colleagues” from the Methods section. The capacity gaps identified in this study are now addressed in the Discussion section*

***Comment 3***

- Row # 219 the full set of codes and themes is presented in supplementary file S2 table not in S1.

***Author Response****:*

*Thank you for pointing this out. This is correctly labelled as Supplementary File S1, S2 Table in the manuscript. S2 table is part of our supplementary file S1.*

Results

***Comment 4***

- Does the analysis presented match the analysis plan? Analysis plan is not applicable
  Are the results clearly and completely presented? yes
  Are the figures (Tables, Images) of sufficient quality for clarity? Table 1: the number and percentage of Job cadre need a review (Total 111 instead of 105)

***Author Response****:*

*We thank the reviewer for pointing this out. The table has now been corrected.*

Conclusions

***Comment 5***

- Are the conclusions supported by the data presented? Yes. However, the sentence in row #507-508 is unclear. There may be missing word.

***Author Response****:*

*We thank the reviewer for pointing this out. We have rephrased the conclusion section for better clarity.*

Are the limitations of analysis clearly described? Yes
Do the authors discuss how these data can be helpful to advance our understanding of the topic under study? yes
Is public health relevance addressed? Yes

Editorial and Data Presentation Modifications?

***Comment 6***

- Authors should consider suggestions for minor revision : This manuscript described information related to intestinal schistosomiasis (*S. mansoni* infection), the following should be changed to *S.mansoni* infection or intestinal schistosmiasis instead of "schistosomiasis" since there is no information about urogenital schistosomiasis:
  - Full Title
  - Short title
  - Row #232, 234, 268, 313, 350, 375
  - themes in tables 2 and 4

***Author Response****:*

*We thank the reviewer for pointing this out. We have changed ‘schistosomiasis’ to ‘intestinal schistosomiasis’ in the full title, short title, and rows #232, 234, 268, 313, 350, 375. We have revised tables 2 and 4 by removing the theme columns.*

Summary and General Comments

This manuscript described gaps in intestinal schistosomiasis case definitions, gaps in case management and patient pathways, the necessity of practical approaches to strengthen health system capacity in three districts in Uganda.
Interesting manuscript which provides information from Health workers that can be applied in other endemic areas. Generalizability of the findings is limited, however there many gaps to be addressed.
