# Supplementary material for: Government health care worker training needs for intestinal schistosomiasis morbidity management
Source: PLoS Negl Trop Dis. 2026 Jun 8;20(6):e0014419. doi: 10.1371/journal.pntd.0014419 (PMC13268167; doi:10.1371/journal.pntd.0014419)
Supplement: Peer review history

# Peer review: Decision Letter 0

**DOI:** 10.1371/journal.pntd.0014419.r001

**Authors:** Qu Cheng, Ran Wang

24 Apr 2026

PNTD-D-26-00523

Government health care worker training needs for schistosomiasis morbidity management

PLOS Neglected Tropical Diseases

Dear Dr. Chami,

Thank you for submitting your manuscript to PLOS Neglected Tropical Diseases. After careful consideration, we feel that it has merit but does not fully meet PLOS Neglected Tropical Diseases’s publication criteria as it currently stands. Therefore, we invite you to submit a revised version of the manuscript that addresses the points raised during the review process.

Please submit your revised manuscript by Jun 23 2026 11:59PM. If you will need more time than this to complete your revisions, please reply to this message or contact the journal office at plosntds@plos.org. When you’re ready to submit your revision, log on to https://www.editorialmanager.com/pntd/ and select the ‘Submissions Needing Revision’ folder to locate your manuscript file.

Please include the following items when submitting your revised manuscript:

* A letter that responds to each point raised by the editor and reviewer(s). You should upload this letter as a separate file labeled ‘Response to Reviewers’. This file does not need to include responses to any formatting updates and technical items listed in the ‘Journal Requirements’ section below.

* A marked-up copy of your manuscript that highlights changes made to the original version. You should upload this as a separate file labeled ‘Revised Manuscript with Track Changes’.

* An unmarked version of your revised paper without tracked changes. You should upload this as a separate file labeled ‘Manuscript’.

If you would like to make changes to your financial disclosure, competing interests statement, or data availability statement, please make these updates within the submission form at the time of resubmission. Guidelines for resubmitting your figure files are available below the reviewer comments at the end of this letter.

We look forward to receiving your revised manuscript.

Kind regards,

Ran Wang, M.D.

Academic Editor

PLOS Neglected Tropical Diseases

Qu Cheng

Section Editor

PLOS Neglected Tropical Diseases

Shaden Kamhawi

co-Editor-in-Chief

PLOS Neglected Tropical Diseases

orcid.org/0000-0003-4304-636XX

Paul Brindley

co-Editor-in-Chief

PLOS Neglected Tropical Diseases

orcid.org/0000-0003-1765-0002

**Journal Requirements:**

Please upload all main figures as separate Figure files in .tif or .eps format. For more information about how to convert and format your figure files please see our guidelines:

https://journals.plos.org/plosntds/s/figures

**Reviewers’ comments:**

Reviewer’s Responses to Questions

**Key Review Criteria Required for Acceptance?**

As you describe the new analyses required for acceptance, please consider the following:

**Methods:**

-Are the objectives of the study clearly articulated with a clear testable hypothesis stated?

-Is the study design appropriate to address the stated objectives?

-Is the population clearly described and appropriate for the hypothesis being tested?

-Is the sample size sufficient to ensure adequate power to address the hypothesis being tested?

-Were correct statistical analysis used to support conclusions?

-Are there concerns about ethical or regulatory requirements being met?

Reviewer #1: No concerns to raise under methodology

Ethical and regulatory requirements adequately met.

Reviewer #2: The manuscript titled "Government health care worker training needs for schistosomiasis morbidity

management" is a good paper that highlight important thematic area about morbidity management of schistosomiasis infection, looking at training and capacity building needs of the health system to be able provide the needed care for those affected. The manuscript is well written with clearly articulated objectives, and it employed the appropriate study design. However, some sentences are too long, and need broken into 2 sentences while some need rephrasing

Reviewer #3: Are the objectives of the study clearly articulated with a clear testable hypothesis stated? Te objectives are stated in different paragraphs of the manuscript: in the introduction and in the study design and participant recruitment. The objectives should be stated in the introduction paragraph and rephrased (What is general objective? and the specific objectives?). A clear testable hypothesis is not required since it is a qualitative study.

Is the study design appropriate to address the stated objectives? YES

Is the population clearly described and appropriate for the hypothesis being tested? There is no need of hypothesis to be tested. Study participants are clearly described

Is the sample size sufficient to ensure adequate power to address the hypothesis being tested? There is no hypothesis to be tested. To get information from a qualitative study the sample size is sufficient. However as stated in the limitations, the workshops did not include all types of health workers in the study area and the generability of the findings is limited.

Were correct statistical analysis used to support conclusions? Statistical analyses are not required since it is a qualitative study.

Are there concerns about ethical or regulatory requirements being met? The study obtained ethics approval and there is no concern identified.

Additional comments:

raw # 203-205: "After the patient pathway...inadequacy to their colleagues" should be removed and used in the discussion paragraph.

Raw # 220 the full set of codes and themes is presented in supplementary file S2 table not in S1.

**********

**Results:**

-Does the analysis presented match the analysis plan?

-Are the results clearly and completely presented?

-Are the figures (Tables, Images) of sufficient quality for clarity?

Reviewer #1: Yes

Reviewer #2: The analysis presented match the analysis plan and the result were clearly and presented in detailed. The tables presented are ok

Reviewer #3: Does the analysis presented match the analysis plan? Analysis plan is not applicable

Are the results clearly and completely presented? yes

Are the figures (Tables, Images) of sufficient quality for clarity? Table 1: the number and percentage of Job cadre need a review (Total 111 instead of 105)

**********

**Conclusions:**

-Are the conclusions supported by the data presented?

-Are the limitations of analysis clearly described?

-Do the authors discuss how these data can be helpful to advance our understanding of the topic under study?

-Is public health relevance addressed?

Reviewer #1: Yes

Reviewer #2: The conclusions were well supported by data presented and the limitation of the analysis were described. In addition, authors explain how their findings can be use in health workers need assessment in other schistosomiasis endemic regions

Reviewer #3: Are the conclusions supported by the data presented? Yes. However, the sentence in raw #507-508 is unclear. There may be missing word.

Are the limitations of analysis clearly described? Yes

Do the authors discuss how these data can be helpful to advance our understanding of the topic under study? yes

Is public health relevance addressed? Yes

**********

**Editorial and Data Presentation Modifications?**

Use this section for editorial suggestions as well as relatively minor modifications of existing data that would enhance clarity. If the only modifications needed are minor and/or editorial, you may wish to recommend “Minor Revision” or “Accept”.

Reviewer #1: Highly relevant topic -manuscript well presented.

Reviewer #2: The manuscript is good and relevant but the authors need to work on some statement, some are too long or need rephrasing for clarity

Reviewer #3: Auhors should consider suggestions for minor revision : This manuscript described information related to intestinal schistosomiasis (S. mansoni infection), the following should be changed to S.mansoni infection or intestinal schistosmiasis instead of "schistosomiasis" since there is no information about urogenital schistosomiasis:

- Full Title

- Short title

- Raw #232, 234, 268, 313, 350, 375

- themes in tables 2 and 4

**********

**Summary and General Comments**

Use this section to provide overall comments, discuss strengths/weaknesses of the study, novelty, significance, general execution and scholarship. You may also include additional comments for the author, including concerns about dual publication, research ethics, or publication ethics. If requesting major revision, please articulate the new experiments that are needed.

Reviewer #1: This manuscript addresses a highly relevant topic of schistosomiasis morbidity, which remains under-reported. The gap between infection-focused interventions (especially mass treatments) and the management of chronic morbidity within routine health systems persists. Using a qualitative needs assessment across three endemic districts in Uganda, the authors provide important insights into health worker knowledge gaps, fragmented patient pathways, and systemic constraints in morbidity management. The topic is timely and aligns well with the evolving global agenda to move beyond preventive chemotherapy toward integrated morbidity management and health system strengthening.

Reviewer #2: The manuscript is relevant and useful for ministry of health of schistosomiasis endemic community that wish to conduct need assessment for management of schisomiasis morbidity at all levels of health health care

Reviewer #3: This manuscript described gaps in intestinal schistosomiasis case definitions, gaps in case management and patient pathways, the necessity of practical approaches to strengthen health system capacity in three districts in Uganda.

Interesting manuscript which provides informations from Health workers that can be happend in other endemic areas. Generalizability of the findings is limited, however there many gaps to be adressed.

**********

PLOS authors have the option to publish the peer review history of their article (what does this mean?). If published, this will include your full peer review and any attached files.

If you choose “no”, your identity will remain anonymous but your review may still be made public.

**Do you want your identity to be public for this peer review?**   For information about this choice, including consent withdrawal, please see our Privacy Policy.

Reviewer #1: No

Reviewer #2: **Yes:**   Akinola Stephen Oluwole

Reviewer #3: No

[NOTE: If reviewer comments were submitted as an attachment file, they will be attached to this email and accessible via the submission site. Please log into your account, locate the manuscript record, and check for the action link "View Attachments". If this link does not appear, there are no attachment files.]

**Figure resubmission:**

While revising your submission, we strongly recommend that you use PLOS’s NAAS tool (https://ngplosjournals.pagemajik.ai/artanalysis) to test your figure files. NAAS can convert your figure files to the TIFF file type and meet basic requirements (such as print size, resolution), or provide you with a report on issues that do not meet our requirements and that NAAS cannot fix.

After uploading your figures to PLOS’s NAAS tool - https://ngplosjournals.pagemajik.ai/artanalysis, NAAS will process the files provided and display the results in the "Uploaded Files" section of the page as the processing is complete. If the uploaded figures meet our requirements (or NAAS is able to fix the files to meet our requirements), the figure will be marked as "fixed" above. If NAAS is unable to fix the files, a red "failed" label will appear above. When NAAS has confirmed that the figure files meet our requirements, please download the file via the download option, and include these NAAS processed figure files when submitting your revised manuscript.

**Reproducibility:**

To enhance the reproducibility of your results, we recommend that authors of applicable studies deposit laboratory protocols in protocols.io, where a protocol can be assigned its own identifier (DOI) such that it can be cited independently in the future. Additionally, PLOS ONE offers an option to publish peer-reviewed clinical study protocols. Read more information on sharing protocols at https://plos.org/protocols?utm_medium=editorial-email&utm_source=authorletters&utm_campaign=protocols

Attachment Submitted filename: PNTD-D-26-00523_reviewer_AO.pdf

# Author comment: Author response to Decision Letter 1

**DOI:** 10.1371/journal.pntd.0014419.r002

8 May 2026

Attachment Submitted filename: Response to reviewers.docx

# Peer review: Decision Letter 1

**DOI:** 10.1371/journal.pntd.0014419.r003

**Authors:** Qu Cheng, Ran Wang

29 May 2026

Dear Dr Chami,

We are pleased to inform you that your manuscript ‘Government health care worker training needs for intestinal schistosomiasis morbidity management’ has been provisionally accepted for publication in PLOS Neglected Tropical Diseases.

Before your manuscript can be formally accepted you will need to complete some formatting changes, which you will receive in a follow up email. A member of our team will be in touch with a set of requests.

Please note that your manuscript will not be scheduled for publication until you have made the required changes, so a swift response is appreciated.

IMPORTANT: The editorial review process is now complete. PLOS will only permit corrections to spelling, formatting or significant scientific errors from this point onwards. Requests for major changes, or any which affect the scientific understanding of your work, will cause delays to the publication date of your manuscript.

Should you, your institution’s press office or the journal office choose to press release your paper, you will automatically be opted out of early publication. We ask that you notify us now if you or your institution is planning to press release the article. All press must be co-ordinated with PLOS.

Thank you again for supporting Open Access publishing; we are looking forward to publishing your work in PLOS Neglected Tropical Diseases.

Best regards,

Ran Wang, M.D.

Academic Editor

PLOS Neglected Tropical Diseases

Qu Cheng

Section Editor

PLOS Neglected Tropical Diseases

Shaden Kamhawi

co-Editor-in-Chief

PLOS Neglected Tropical Diseases

orcid.org/0000-0003-4304-636XX

Paul Brindley

co-Editor-in-Chief

PLOS Neglected Tropical Diseases

orcid.org/0000-0003-1765-0002

***********************************************************

p.p1 {margin: 0.0px 0.0px 0.0px 0.0px; line-height: 16.0px; font: 14.0px Arial; color: #323333; -webkit-text-stroke: #323333}span.s1 {font-kerning: none

Reviewer’s Responses to Questions

**Key Review Criteria Required for Acceptance?**

As you describe the new analyses required for acceptance, please consider the following:

**Methods**

-Are the objectives of the study clearly articulated with a clear testable hypothesis stated?

-Is the study design appropriate to address the stated objectives?

-Is the population clearly described and appropriate for the hypothesis being tested?

-Is the sample size sufficient to ensure adequate power to address the hypothesis being tested?

-Were correct statistical analysis used to support conclusions?

-Are there concerns about ethical or regulatory requirements being met?

Reviewer #3: The objectives of the study are clearly articulated and revised according to my suggestions

The study designed is appropriate to adress the objectives

The study population is clearly described

To get information from a qalitative study, the sample size is sufficient

Statistical analyses are not required since it is qualitative study

No concern identified about ethical or regulatory requirement

**********

**Results**

-Does the analysis presented match the analysis plan?

-Are the results clearly and completely presented?

-Are the figures (Tables, Images) of sufficient quality for clarity?

Reviewer #3: All recommendation for minor changes are adressed

**********

**Conclusions**

-Are the conclusions supported by the data presented?

-Are the limitations of analysis clearly described?

-Do the authors discuss how these data can be helpful to advance our understanding of the topic under study?

-Is public health relevance addressed?

Reviewer #3: the responses to these questions are: YES

**********

**Editorial and Data Presentation Modifications?**

Use this section for editorial suggestions as well as relatively minor modifications of existing data that would enhance clarity. If the only modifications needed are minor and/or editorial, you may wish to recommend “Minor Revision” or “Accept”.

Reviewer #3: (No Response)

**********

**Summary and General Comments**

Use this section to provide overall comments, discuss strengths/weaknesses of the study, novelty, significance, general execution and scholarship. You may also include additional comments for the author, including concerns about dual publication, research ethics, or publication ethics. If requesting major revision, please articulate the new experiments that are needed.

Reviewer #3: (No Response)

**********

PLOS authors have the option to publish the peer review history of their article (what does this mean?). If published, this will include your full peer review and any attached files.

If you choose “no”, your identity will remain anonymous but your review may still be made public.

**Do you want your identity to be public for this peer review?**   For information about this choice, including consent withdrawal, please see our Privacy Policy.

Reviewer #3: No

# Editor report: Acceptance letter

**DOI:** 10.1371/journal.pntd.0014419.r004

**Authors:** Qu Cheng, Ran Wang

Dear Dr Chami,

We are delighted to inform you that your manuscript, "Government health care worker training needs for intestinal schistosomiasis morbidity management," has been formally accepted for publication in PLOS Neglected Tropical Diseases.

We have now passed your article onto the PLOS Production Department who will complete the rest of the publication process. All authors will receive a confirmation email upon publication.

The corresponding author will soon be receiving a typeset proof for review, to ensure errors have not been introduced during production. Please review the PDF proof of your manuscript carefully, as this is the last chance to correct any scientific or type-setting errors. Please note that major changes, or those which affect the scientific understanding of the work, will likely cause delays to the publication date of your manuscript. Note: Proofs for Front Matter articles (Editorial, Viewpoint, Symposium, Review, etc...) are generated on a different schedule and may not be made available as quickly.

Soon after your final files are uploaded, the early version of your manuscript will be published online unless you opted out of this process. The date of the early version will be your article’s publication date. The final article will be published to the same URL, and all versions of the paper will be accessible to readers.

For Research Articles, you will receive an invoice from PLOS for your publication fee after your manuscript has reached the completed accept phase. If you receive an email requesting payment before acceptance or for any other service, this may be a phishing scheme. Learn how to identify phishing emails and protect your accounts at https://explore.plos.org/phishing.

Thank you again for supporting open-access publishing; we are looking forward to publishing your work in PLOS Neglected Tropical Diseases.

Best regards,

Shaden Kamhawi

co-Editor-in-Chief

PLOS Neglected Tropical Diseases

Paul Brindley

co-Editor-in-Chief

PLOS Neglected Tropical Diseases
